# Long-Term Performance of Modern Coronary Sinus Leads in Cardiac Resynchronization Therapy

**DOI:** 10.1016/s0972-6292(16)30752-5

**Published:** 2014-05-25

**Authors:** Jan Steffel, Anja Hurlimann, Christoph Starck, Nazmi Krasniqi, Susann Schmidt, Thomas F Luscher, Firat Duru, Frank Ruschitzka, Johannes Holzmeister, David Hurlimann

**Affiliations:** 1 Department of Cardiology, University Hospital Zurich; 2 Department of Cardiac Surgery, University Hospital Zurich

**Keywords:** Coronary Sinus Leads, Cardiac Resynchronization Therapy

## Abstract

**Background:**

Cardiac resynchronization therapy (CRT) has become an important pillar of contemporary heart failure therapy. The efficacy of CRT, however, critically relies on proper LV lead placement and performance, which is why data regarding the long-term performance of CS leads are of considerable interest. Available studies are limited by a restricted variety of lead vendors, earlier lead models and / or very short follow-up periods. In the current study, we therefore investigated the long-term performance of modern LV leads in a large "real world" cohort of patients undergoing CRT implantation.

**Methods and Results:**

All 193 patients who had successfullyundergone CRT implantation at the University Hospital Zurich between September 2003 and January 2010 were included in the study. An overall stable course of stimulation energy was observed over time; neither ischemic etiology, lead configuration, or severely reduced EF had an influence on the evolution of energy thresholds over time. Interestingly, patients with a high energy threshold at baseline experienced a significant reduction during follow-up. In contrast, a significant drop in impedance was seen following implantation, followed by a steady course for the rest of the observation period. Only 15 patients (9.7%) showed an impedance > 1000 Ohm at any time during their follow-up. Seven lead dislocations were observed during follow up.

**Conclusion:**

The current comprehensive analysis of long-term performance of modern coronary sinus leads demonstrates excellent stability, performance and safety. These data may have important implications for physicians involved in biventricular pacemaker implantations and in the follow-up care of these patients.

## Introduction

Cardiac resynchronization therapy (CRT) has become an important pillar of contemporary heart failure therapy due to the impressive reduction in morbidity and mortality observed in large-scale clinical trials [1-3]. Several myocardial diseases are associated with changes in cardiac structure and function resulting in regions of early and late left ventricular (LV) contraction, ultimately leading to intra- and interventricular dyssynchrony and impaired left-ventricular contractile function. In CRT, biventricular pacing re-synchronizes the left ventriclevia an LV lead placed in a tributary of the coronary sinus (CS) in addition to the standard right atrial and right ventricular leads, resulting in an increase in cardiac output and reverse left ventricular remodeling [4].

The efficacy of biventricular pacing critically relies on proper LV lead placement and performance. The former, however, is not infrequently challenging; indeed, lead placement at anatomically optimal sites for effective LV resynchronization sometimes requires accepting high capture thresholds due to suboptimal lead contact or presence of scar tissue. As such, the possibility of late lead failure due to rising thresholds, increased battery drain,and, eventually even loss of biventricular pacing is of concern. Data regarding the long-term performance of CS leads, especially in relation to baseline characteristics at the time of implantation, are therefore of considerable interest. So far, few studies have investigated the behavior of LV leads in CRT patients; moreover, these series were limited by a restricted variety of lead vendors, earlier lead models and / or very short follow-up periods [5-10]. In the current study, we therefore investigated the long-term performance of modern LV leads in a large "real world" cohort of patients undergoing CRT implantation.

## Methods

### Study population and CRT implantation procedure

All 193 patients who had undergone successful CRT implantation including placement of a coronary sinus left ventricular lead at the University Hospital Zurich between September 2003 and January 2010 were included in the study (patients implanted by J.H. and D.H.). Patients in whom transvenous LV lead placement was not possible as well as patients with epicardial LV leads were excluded from the study. For analyses of follow-up parameters, patient files at our pacemaker clinic were reviewed.

Biventricular pacemakers / ICDs and leads from Biotronik, Guidant / Boston Scientific, Medtronic and St. Jude Medical were implanted (Table 1). The implantation procedure was performed under local anaesthesia using standard techniques. For implantation of the left ventricular (LV) lead, the coronary sinus (CS) was intubated with a CS sheath, followed by a CS venogram to evaluate coronary vein anatomy. Implantation into a lateral or postero-lateral vein in the midventricular region of the LV was attempted whenever possible. Real-time measurements of LV lead stimulation threshold, impedance and R-wave sensing was performed using a Medtronic analyzer system (Medtronic 8900, Medtronic Inc, Minneapolis MN, USA). The energy threshold required for reproducible successful LV capture is presented as stimulation energy derived from the following formula: E = V2 x t / R (E = energy [μJ]; V = voltage [V]; t = stimulation pulse width [s]; R = impedance [Ohm]). Echocardiography, including left ventricular scar assessment was performed according to standard protocols (Philips iE33 or GE Vivid 7).

### Baseline and follow-up measurements

LV lead measurements at implantation were taken in the operating room following successful lead placement. Baseline measurements were taken prior to hospital discharge (one day following device implantation in most patients). Further measurements were recorded when patients presented for scheduled follow-up visits to our pacemaker clinic or in case of unplanned consultations.

### Statistics

Statistical analyses were performed using SPSS 17.0 (SPSS Inc., Chicago, IL) and GraphPad Prism 4.0 (GraphPad Software, San Diego, USA). Unpaired continuous and categorical variables were compared using Student's t-test and Chi-square test, respectively. Intraindividual development of variables (energy threshold, impedance and sensing at baseline and follow-up) were compared using paired Student's t-test. Comparisons of > 2 groups were performed using an ANOVA test with Bonferroni correction in case of significant results. A p-value < 0.05 was considered significant.

## Results

### Baseline characteristics and follow-up

Baseline clinical, echocardiographic and implantation characteristics are summarized in Table 1. Clinical and echocardiographic findings are those typical of a cohort of patients with chronic heart failure. Devices and leads from all four manufacturers were implanted; however, both leads and devices from Guidant / Boston Scientific were only used in a minority of cases.

**Figure 1:**
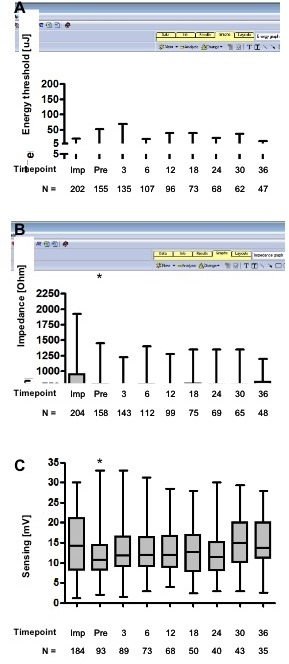
Long-term development of left ventricular leads energy threshold (A), impedance (B) and sensing (C). Mean, standard deviation and range are shown.

Median follow-up of all patients with at least one follow-up visit at our center was 24 months. The evolution of coronary lead stimulation energy threshold, impedance and R-wave sensing is shown in Figure 1. For stimulation energy, an overall stable course was observed over time. In contrast, a significant drop in impedanc was seen following implantation, followed by a steady course for the remainder of the observation period. Similarly, albeit to a lesser degree, a drop in the sensed R-wave amplitude occurred following implantation, with no significant change over the ensuing months.

### Evolution of LV lead threshold

Selected subgroup comparisons of energy threshold, impedance, and R-wave sensing at baseline (pre-discharge) and during follow-up (12-15 months) are shown in Table 2. In order to perform a valid analysis, only patients with both baseline and follow-up values were compared. No statistically significant differences in energy threshold, impedance or R-wave sensing were observed at baseline and at 12 months follow-up when patients without pre-discharge and follow-up measurements, respectively, were excluded (data not shown).

Overall, no significant difference in average energy threshold was observed during 1-year follow-up as compared to baseline (Table 2). Neither ischemic etiology, gender,lead configuration, nor severely reduced EF had an influence on the development of energy thresholds over time (data not shown). A marginally significant effect of the lead manufacturer most likely occurred as a result of chance due to the low number of leads implanted from one vendor. Interestingly, patients with a high energy threshold at baseline (defined as > 1μJ) experienced a significant reduction during follow-up; in contrast, those with a lower threshold at baseline demonstrated a rise in threshold over time.

Overall, 67 patients (35.4%) had a high LV lead threshold at implantation, which was particularly prevalent in patients with an LV scar (20 / 38 patients, 53%; p=0.012 vs. no scar). During follow-up, 22% of patients (33/152) had a very high maximal LV threshold (defined as > 3μJ). Patients with a threshold > 1μJ at implantation had a higher chance of going on to a threshold ≥ 3μJ at any time during follow up as compared to patients with a threshold < 1μJ (34% vs. 16%, p=0.006).

### Evolution of LV lead threshold

During follow-up, no significant change in impedance from pre-discharge values was observed for the overall cohort (Tab. 2). As expected, impedance was higher in bipolar leads, which did not change during follow up. Also patients with a low EF (< 30%) demonstrated slightly lower LV lead impedance. Other baseline characteristics including coronary heart disease, gender,lead configuration, and LV scar did not significantly affect LV lead impedance at baseline or at follow-up.

Patients with impedance values > 1000 Ohm at the time of implantation experienced a significant drop until the time of hospital discharge (Figure 1). Indeed, only 28% of patients with an impedance > 1000 Ohm at implantation still had a high impedance at pre-discharge, while it had normalized in the remaining 72% (data not shown). Only 15 patients (9.7%) showed an impedance > 1000 Ohm at any time during their follow-up; no baseline characteristics, including a high impedance after implantation, was more frequently found in this subgroup of patients.

### Evolution of LV lead R-wave sensing

On average, left ventricular R-wave sensing improved slightly during follow up (11.5 +/- 6.3 mV vs. 13.5 +/- 6.2 mV, p=0.007). This was particularly pronounced in patients with non-ischemic heart disease, in those with a bipolar lead, and in those with no scar or a scar outside the prime target region for LV lead placement (while patients with a scar in the lateral or posterior LV segments experienced a decrease in R-wave sensing over time).

### Lead Dislocations

A total of 7 cases of LV-lead dislocations (3.6%) were observed during follow-up. The median time to dislocation was 111 days (range 10 - 283 days). Six cases were macro-dislocations necessitating lead revision. In one case a micro-dislocation leading to considerably elevated stimulation thresholds was observed, which was managed conservatively (i.e., without re-do operation). No significant adverse events, including arrhythmic events, were observed as a result of dislocated leads.

## Discussion

Optimal lead placement is of paramount importance for a favorable outcome in CRT [11] Indeed, suboptimal lead positioning may be one of the key factors involved in the lack of response to CRT, which can be observed in as many as 30% of CRT recipients [11-13]. Implantation into a lateral or postero-lateral vein is therefore attempted whenever possible, as this has been associated with the highest likelihood of clinical and echocardiographic improvement [4] However, high LV capture thresholds as well as high impedance values are not infrequently encountered at those otherwise optimal sites. In these situations, considerable uncertainty regarding the future behavior of these leads is imminent during implantation. As a result, a well-suited LV pacing site may be abandoned for another, potentially less suitable position in order to achieve a lower energy threshold or impedance.

Our current comprehensive analysis of the long-term behavior of modern coronary sinus leads provides several findings which may be of value for physicians implanting and/or managing patients with CRT devices. We demonstrate that most patients with a high energy threshold during implantation normalize over time, while a third (34%) increases to very high levels. Neither ischemic etiology, lead configuration, severely reduced EF nor presence of a scar had a significant influence on the future development of energy thresholds. This is in contrast to previous studies, in which a pronounced increase over time was observed in particular in patients with unipolar leads [9] In the latter study, only patients undergoing LV lead implantation during 1999-2003 were included, and, consequently, only earlier generation leads from two vendors were used (Guidant Easytrak 1 and Medtronic Attain 2187). A similar increase in LV capture threshold was observed in patients undergoing CRT during earlier years (1994 - 2002) [7,10]. In contrast, the current study included patients from 2003 - 2010 with modern, mainly bipolar leads, with improved characteristics from all four major manufacturers, which likely contributed to the more favorable results. The only subgroup to demonstrate an increased likelihood for a high threshold at implantation as well as during follow-up were those with a scar in the inferior, lateral or posterior region. Interestingly, Biotronik LV leads showed a significant decrease in LV capture thresholds over time, which was not observed to this degree with other manufacturers. Performance of these particular leads has rarely been reported so far, and further studies are warranted to substantiate these findings.

Similar to the capture thresholds, high impedance values at implantation normalized in most patients (82%) during the ensuing hours to days, and only a minority increased to higher levels over time. These findings indicate that in an anatomically optimal position, the mere presence of an elevated impedance (i.e., > 1000 Ohm) should not in itself prompt for lead relocation to a potentially less suitable LV pacing site. Indeed, no baseline variables including the presence of coronary heart disease, lead configuration, and LV scar were predictive of an elevated LV lead impedance at baseline or during follow-up. As expected, LV lead impedance was slightly higher in bipolar leads, which did not change during follow up. There was no difference between the leads of different manufacturers.

A small but significant increase in LV sensing values was observed during follow-up, which was most pronounced in patients with non-ischemic cardiomyopathy, bipolar leads, and better (> 30%) LV ejection fraction. In contrast, and as expected, R-wave sensing decreased in the case of scar tissue in the target area of LV lead implantation (lateral or posterior wall segments). In CRT, LV sensing is considered of minor value, as ventricular sensing is usually performed via the right ventricular electrode, in particular in CRT-ICD devices. However, our data on the development of LV sensing values confirm the overall stable course indicated by stable capture thresholds and impedance values of modern LV leads, potentially providing an option as primary pacing leads (instead of an RV lead e.g. in patients with reconstructed tricuspid valve or bioprosthesis).

In contrast to right ventricular pacing leads, LV leads typically do not have a fixation mechanism (screw/tines) but are passively held in place making them more prone to dislocation, which may result in alterations in pacing parameters. In general, elevated pacing thresholds result in faster battery depletion, leading to more frequent device replacements (associated with potential peri-interventional risks) as well as increased costs. While the limited amount of patients with very long-term follow-up (i.e. until the time of generator exchange) precluded a meaningful and comprehensive analysis in this regard, elevated LV thresholds will likely be associated with earlier battery depletion also in our cohort. Overall, only 7 lead dislocations (3.6%) were observed during the follow-up in our cohort (6 of which necessitated lead revision),comparable to the recent Euroheart Survey, highlighting the excellent stability, performance and safety of today's LV leads [14] In one case a micro-dislocation leading to considerably elevated stimulation thresholds was observed, which was managed conservatively.

Our study is limited by its retrospective design; as such, capture thresholds were measured at varying pulse width, for which we corrected by calculation of stimulation energy thresholds. Although this may render direct transferability of our data difficult, it represented the only valid way to account for these inherent differences. Further limitations include the fact that leads from one vendor (Guidant / Boston Scientific) were only used in a minority of patients.

## Conclusions

The current comprehensive analysis of the long-term performance of modern coronary sinus leads revealed a stable course of these leads over time. An elevated energy capture threshold at implantation was associated with an increased likelihood of proceeding to very high thresholds during follow-up, which was the case in 34% of these patients. These data may have important implications for physicians involved in biventricular pacemaker implantations and in the follow-up care of these patients.

## Figures and Tables

**Table 1:**
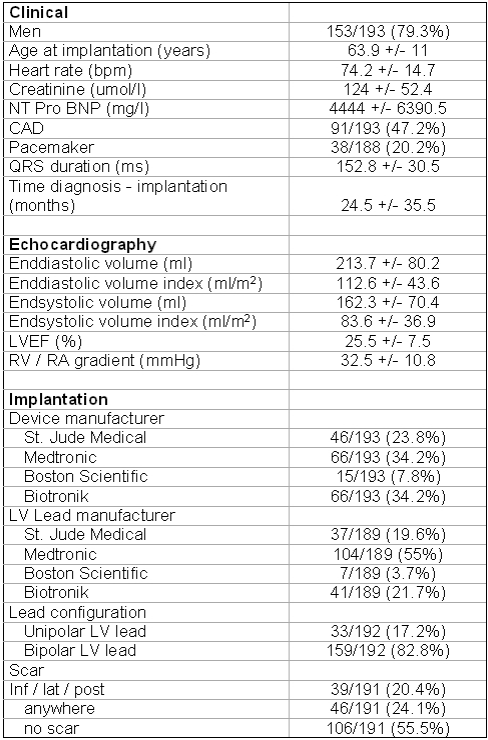
Baseline characteristics at implantation

Continuous and categorical variables are presented as mean +/- standard deviation and number (%), respectively.

**Table 2:**
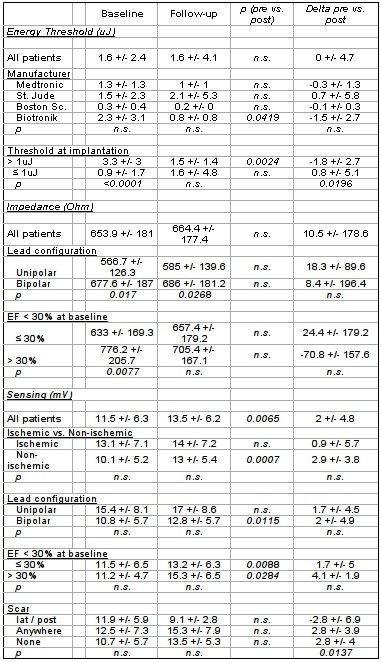
Energy threshold, impedance and R-wave sensing at baseline (pre-discharge) and at 12 (-15) months follow up of the entire cohort and for selected subgroups.

Data are presented as mean +/- standard deviation. n.s. = not significant.
